# Neurohumoral Dysregulation in Acute Myocardial Infarction With Chronic Kidney Disease: Implications for Prognosis and Management

**DOI:** 10.7759/cureus.93692

**Published:** 2025-10-02

**Authors:** Oksana Polianska, Victor Tashchuk, Olha Hulaha, Valentyna Olinchuk, Inna Moskaliuk, Yevhen Kushnir

**Affiliations:** 1 Department of Internal Medicine, Physical Rehabilitation and Sport Medicine, Bukovinіan State Medical University, Chernivtsi, UKR; 2 Department of Internal Medicine, Physical Rehabilitation and Sports Medicine, Bukovinian State Medical University, Chernivtsi, UKR; 3 Department of Cardiology, Clinic Optima Medycyna, Opole, POL; 4 Department of Internal Medicine, Bukovinian State Medical University, Chernivtsi, UKR; 5 Department of Family Medicine, University of Opole, Opole, POL; 6 Department of Internal Medicine, St. Barnabas Hospital, New York, USA

**Keywords:** aldosterone, angiotensin-converting enzyme, chronic kidney disease, heart failure, myocardial infarction, neurohumoral imbalance, renin-angiotensin-aldosterone system

## Abstract

Background

Acute myocardial infarction (AMI) and chronic kidney disease (CKD) often coexist, and both are associated with neurohumoral imbalances that may worsen cardiovascular outcomes. Reduced kidney function can contribute to arterial stiffness and activation of the renin-angiotensin-aldosterone system (RAAS), which promotes vasoconstriction, sodium and water retention, myocardial remodeling, and fibrosis, mechanisms that exacerbate both cardiac and renal dysfunction. Endothelial injury is also common in these patients, and von Willebrand factor (vWF) serves as a recognized marker of endothelial dysfunction, contributing to thrombotic risk and microvascular impairment.

Objective

To explore the cross-sectional associations between renal function and the biomarkers aldosterone, angiotensin-converting enzyme (ACE), atrial natriuretic peptide (ANP), and vWF in patients with AMI and heart failure.

Methods

In this cross-sectional study, 106 patients hospitalized for AMI and heart failure were stratified by estimated glomerular filtration rate (GFR) into Group 1 (≤90 mL/min) and Group 2 (>90 mL/min). While CKD is conventionally defined as GFR <60 mL/min, the ≤90 mL/min cutoff was chosen to evaluate biomarker variation across a broader renal function range, including early decline. All eligible patients from the study period were included; no formal power calculation was performed. Neurohumoral markers were measured via enzyme-linked immunosorbent assay (ELISA). Heart failure was an inclusion criterion for all participants.

Results

Baseline characteristics were comparable between groups. Aldosterone and ACE were higher in patients with GFR ≤90 mL/min (p-values 0.01-0.05; below the prespecified α = 0.01). ANP and vWF did not differ between groups, which may reflect limited sensitivity to early renal function differences or other physiological influences.

Conclusion

The observed association between reduced GFR and elevated aldosterone and ACE suggests RAAS-related neurohumoral activation in AMI patients with heart failure. The lack of difference in ANP and vWF highlights potential marker-specific limitations. Targeting RAAS and other relevant pathways may offer therapeutic benefits for improving outcomes in this population; however, such approaches require confirmation in prospective interventional trials. These findings are exploratory and hypothesis-generating, underscoring the need for larger, longitudinal, and interventional studies to clarify the prognostic and therapeutic implications.

## Introduction

Over the past two decades, the global mortality rate for cardiovascular diseases has decreased, yet the incidence of and mortality from acute myocardial infarction (AMI) have continued to rise, particularly among patients with coexisting chronic kidney disease (CKD). CKD has long been recognized as a significant risk factor for cardiovascular morbidity and mortality, with studies showing that even mild renal dysfunction (elevated serum creatinine levels of 124-200 µmol/L) is linked to a 40% increase in cardiovascular events. More severe reductions in renal function (glomerular filtration rate [GFR] <60 mL/min) correlate with a 50% higher risk of cardiovascular mortality [1,2].

The interplay between CKD and AMI is complex, as both conditions are characterized by neurohumoral imbalances, notably activation of the renin-angiotensin-aldosterone system (RAAS), the sympathetic nervous system, endothelin pathways, and other vasoconstrictive mediators. These mechanisms contribute to fluid retention, left ventricular remodeling, and worsening heart failure (HF). CKD is associated with increased arterial stiffness even in the early stages, a factor thought to be a key mediator of the heightened cardiovascular risk [3].

Clinical studies, particularly those conducted in controlled animal models with supportive but more limited human data, have shown that elevated aldosterone and other mineralocorticoids can increase the infarct area and play an important role in post-myocardial infarction (MI) neovascular formation within the first week after MI [4]. While experimental models provide strong mechanistic evidence for these effects, the strength of the evidence in human populations is less definitive and warrants further investigation. Whether these effects are equally pronounced in AMI patients with CKD remains uncertain, and RAAS activation in this subpopulation should be regarded as a suspected rather than proven mechanism.

The inclusion of atrial natriuretic peptide (ANP) and von Willebrand factor (vWF) in this study provides a broader evaluation of neurohumoral and vascular involvement. ANP is a key regulator of fluid balance and cardiac preload, while vWF is a marker of endothelial dysfunction, which is relevant given the vascular injury and thrombotic risk associated with both AMI and CKD. However, these biomarkers can also be influenced by comorbidities such as hypertension and HF, which may confound their interpretation.

The objective of this study was to investigate the neurohumoral changes associated with CKD in patients with AMI, focusing on aldosterone, angiotensin-converting enzyme (ACE), ANP, and vWF. Given the paucity of comparative data on these biomarkers across stratified renal function in AMI, our analysis aims to address this gap. Elevated aldosterone and ACE levels in patients with reduced kidney function suggest that neurohumoral dysregulation may play a significant role in disease progression. Targeting these pathways could improve patient outcomes [5], but prospective interventional trials are needed to confirm therapeutic benefit.

## Materials and methods

This was a hospital-based, cross-sectional observational study conducted at the Department of Internal Medicine, Physical Rehabilitation, and Sports Medicine of Bukovinian State Medical University (Chernivtsi, Ukraine). Data were collected from 15 January 2007 to 20 December 2011 as part of the institutional project “Clinical-pathogenetic and neuromessenger mechanisms of HF development and manifestation in the context of acute coronary syndrome and stable angina with optimization of treatment strategy and identification of prognostic predictors” (State Registration No. 01.U004953). Ethical approval was granted by the Commission on Biomedical Ethics of Bukovinian State Medical University.

Although patient accrual occurred in 2007-2011, this prespecified 2024 re-analysis is hypothesis-generating and centers on neurohumoral associations (aldosterone, ACE, ANP, vWF) rather than treatment outcomes. RAAS-related and endothelial mechanisms remain integral to AMI/CKD pathophysiology [6]; accordingly, while these biomarker insights are clinically pertinent, evolving standards of care limit direct extrapolation, and results should be interpreted within contemporary practice. Given the cross-sectional design, analyses are descriptive/associative and do not support causal or prognostic inference.

Inclusion criteria were adults aged 40-60 years admitted to the inpatient cardiology unit with AMI and clinical HF based on European Society of Cardiology (ESC) guidance [6]. To limit etiologic heterogeneity, CKD cases were restricted to chronic pyelonephritis in remission.

Exclusion criteria were non-ST-segment elevation myocardial infarction (NSTEMI), active pyelonephritis or other infections, diabetes mellitus or other systemic endocrine disorders, chronic liver disease, malignancy, autoimmune disease, corticosteroid or immunosuppressive therapy within six months before admission, and incomplete clinical or laboratory data.

A total of 106 eligible patients were enrolled. At admission, estimated GFR was calculated using the Cockcroft-Gault formula [7]:

\[ \text{GFR} = \frac{(140 - \text{age}) \times \text{weight (kg)} \times K}{\text{serum creatinine} \; (\mu\text{mol/L})} \]

\[ K = \begin{cases} 1.23 & \text{for men} \\ 1.04 & \text{for women} \end{cases} \]

Patients were stratified a priori to explore biomarker trends across a spectrum of renal function: Group 1 (≤90 mL/min; n = 54) and Group 2 (>90 mL/min; n = 52). We note that 60-89 mL/min typically reflects mildly decreased GFR and is not diagnostic of CKD without additional markers; the ≤90 mL/min threshold was not used to diagnose CKD but to sample early biomarker variation across broader renal function. Grouping was independent of CKD status. CKD history (chronic pyelonephritis in remission) was recorded when present; albuminuria and structural markers were not systematically available and are acknowledged as limitations.

HF was an inclusion criterion for all participants. Diagnosis followed ESC (2007) criteria [6], incorporating compatible symptoms/signs (e.g., pulmonary rales, peripheral edema, elevated jugular venous pressure) with echocardiographic evidence of reduced ejection fraction (≤50%). BNP/NT-proBNP values were included when available; natriuretic peptides were not uniformly measured.

All patients underwent ECG, echocardiography, and blood-pressure monitoring. Within 24 hours of admission, venous blood was drawn. Serum aldosterone and ANP were measured using immunoassays per manufacturer instructions; ACE was reported as enzymatic activity (µmol/min/L); vWF was measured by immunoassay. Assays were run in duplicate with internal quality controls; kits/reagents were stored per manufacturer specifications with temperature monitoring. Inter- and intra-assay variabilities were recorded, and laboratory personnel were blinded to the GFR group when feasible.

Data were entered into Microsoft Excel (version 11.5612.5703; Microsoft, Redmond, WA) and analyzed using Statgraphics Plus 5.1 (Statistical Graphics Corp., Warrenton, VA, USA). Double-entry and random audits were performed for data QA. Distribution normality was assessed by the Kolmogorov-Smirnov test. Normally distributed variables are presented as mean ± SD and compared using Student’s t-test; non-normal variables are presented as median (IQR) and compared using the Mann-Whitney U-test. Categorical variables were assessed using the chi-square (χ²) test. Correlations with GFR were examined using Pearson (normal) or Spearman (non-normal) methods. For primary between-group biomarker comparisons, statistical significance was prespecified at p <0.01; values 0.01-0.05 are reported as nominal. Given the exploratory nature and multiple biomarkers assessed, uncorrected p-values are reported, and the risk of Type I error is acknowledged. No multivariable adjustment was performed.

A formal a priori power calculation was not performed; the sample size (n = 106) reflects all eligible cases during the accrual period and was intended to detect moderate between-group differences in primary biomarkers. Although collected in 2007-2011, this focused 2024 analysis emphasizes neurohumoral profiles in AMI with varying renal function. The underlying RAAS-related mechanisms and clinical context remain relevant to contemporary practice; nevertheless, changes in standards of care since the accrual period are acknowledged as a limitation.

Limiting enrollment to ages 40-60 years and to CKD secondary to pyelonephritis in remission reduced etiologic and age-related heterogeneity but may limit external validity to older, higher-risk patients and other CKD etiologies.

## Results

A total of 106 patients with AMI were enrolled and stratified by renal function into two analytic groups to examine early biomarker variation across a spectrum of kidney function: lower GFR (≤90 mL/min, n = 54) and preserved GFR (>90 mL/min, n = 52). We note that 60-89 mL/min typically reflects mildly decreased GFR and is not diagnostic of CKD without additional markers; thus, the ≤90 mL/min cutoff is used here for exploratory stratification rather than CKD definition.

Baseline demographic and clinical characteristics were comparable between groups. No statistically significant differences were observed for age, sex distribution, body mass index (BMI), prevalence of hypertension, smoking history, or prior MI (all p > 0.05). Full baseline characteristics are shown in Table 1.

**Table 1:** Baseline Characteristics of Study Participants Values are presented as mean ± SD or percentages. p-Values for continuous variables were calculated using the Mann-Whitney U-test (or Student’s t-test if normally distributed) and chi-square for categorical variables. No multivariable adjustment was performed; baseline comparability was assessed univariately, and potential residual confounding is acknowledged. No statistically significant differences were observed between groups (p > 0.05).

Characteristic	Group 1 (GFR ≤ 90 mL/min)	Group 2 (GFR > 90 mL/min)	p-Value
Number of patients (n)	54	52	–
Age (years, mean ± SD)	51.6 ± 3.9	51.3 ± 4.0	0.74
Sex (Male/Female)	32/22	31/21	0.91
Hypertension, n (%)	33 (61.1%)	31 (59.6%)	0.87
History of smoking, n (%)	21 (38.9%)	19 (36.5%)	0.80
Prior myocardial infarction, n (%)	8 (14.8%)	7 (13.5%)	0.85
BMI (kg/m², mean ± SD)	26.4 ± 1.9	26.2 ± 1.8	0.68

Distribution normality was assessed using the Kolmogorov-Smirnov test. Normally distributed variables are presented as mean ± SD; non-normal variables are presented as median (IQR). Patients with lower GFR (≤90 mL/min) had higher aldosterone and ACE than those with preserved GFR (>90 mL/min) (both p < 0.05; nominal), not meeting the prespecified p < 0.01 threshold. ANP did not differ between groups. vWF central tendency values were numerically higher in the preserved-GFR group; however, this difference did not reach statistical significance, consistent with a small effect size and variability. Detailed comparisons are presented in Table 2.

**Table 2:** Neurohumoral Marker Levels by Renal Function Group Between-group p-values were calculated using the Mann-Whitney U-test. Statistical significance was prespecified at p < 0.01. Reference intervals (for context): Aldosterone ~100-400 pmol/L; ACE ~8-52 µmol/min/L; ANP ~20-77 pmol/L; vWF ~0.5-1.5 IU/mL. ^†^Nominal (0.01 ≤ p < 0.05); not significant at the prespecified p < 0.01 threshold. GFR, glomerular filtration rate; ACE, angiotensin-converting enzyme; ANP, atrial natriuretic peptide; vWF, von Willebrand factor.

Marker	Group	Mean ± SD	Median (IQR)	p-Value
Aldosterone (pmol/L)	GFR ≤ 90	251.54 ± 9.34	251.2 (245.7-258.6)	<0.05^†^
	GFR > 90	236.24 ± 8.83	236.5 (228.4-242.8)	
ACE (µmol/min/L)	GFR ≤ 90	72.83 ± 2.43	73.0 (71.2-74.4)	<0.05^†^
	GFR > 90	65.28 ± 3.81	65.5 (62.0-68.1)	
ANP (pmol/L)	GFR ≤ 90	28.5 ± 3.2	28.4 (26.0-30.7)	>0.05
	GFR > 90	27.8 ± 2.9	27.9 (25.2-30.1)	
vWF (IU/mL)	GFR ≤ 90	1.06 ± 0.13	1.04 (0.97-1.14)	>0.05
	GFR > 90	1.20 ± 0.07	1.21 (1.15-1.26)	

Correlation analysis showed weak-to-moderate inverse associations between GFR and aldosterone (r = −0.42, p < 0.01) and between GFR and ACE (r = −0.36, p < 0.01). For non-normally distributed variables (ANP, vWF), Spearman analyses yielded similar magnitudes and remained non-significant (ANP: ρ = −0.10, p = 0.31; vWF: ρ = +0.11, p = 0.28). These findings indicate associations rather than causal effects (see Table 3). Given the exploratory nature and multiple biomarkers tested, we report uncorrected p-values and acknowledge the risk of Type I error.

**Table 3:** Correlation Between GFR and Neurohumoral Markers Correlation coefficients are reported as r/ρ: Pearson (r) for normally distributed variables and Spearman (ρ) for non-normal variables. Statistical significance was prespecified at α = 0.01; exact p-values are shown. GFR, glomerular filtration rate; ACE, angiotensin-converting enzyme; ANP, atrial natriuretic peptide; vWF, von Willebrand factor.

Marker	Correlation with GFR (r/ρ)	p-Value
Aldosterone (pmol/L)	r = −0.42	<0.01
ACE (µmol/min/L)	r = −0.36	<0.01
ANP (pmol/L)	ρ = −0.10	0.31
vWF (IU/mL)	ρ = +0.11	0.28

Patients with lower GFR (≤90 mL/min) exhibited higher aldosterone and ACE levels than those with preserved GFR (>90 mL/min) (both p < 0.05; not crossing α = 0.01). ANP did not differ, and vWF was numerically higher in the preserved-GFR group but not statistically significant. These findings are associative and non-prognostic, and should be interpreted in light of the exploratory design and the absence of multivariable adjustment.

## Discussion

This study examines the associations between renal function and neurohumoral markers in patients with AMI. Using GFR strata as an exploratory framework, patients with lower GFR (≤90 mL/min) had higher aldosterone and ACE levels than those with preserved GFR (>90 mL/min). Between-group differences were nominal (p ≈ 0.01-0.05) and did not meet our prespecified threshold (p < 0.01); accordingly, these findings should be interpreted as associative rather than causal. In keeping with KDIGO, a GFR of 60-89 mL/min/1.73 m² reflects a mild decrease and is not diagnostic of CKD without additional markers [8]. Our choice of a >90 mL/min comparator was deliberate to sample a broader renal function spectrum and explore potential early biomarker gradients. We acknowledge that the ≤90 mL/min cutoff may include participants with normal GFR, potentially diluting between-group differences; this motivates future studies using KDIGO-aligned strata (>90, 60-89, <60 mL/min/1.73 m²) with adequate power.

The pattern of higher aldosterone and ACE in lower GFR strata is directionally consistent with RAAS involvement in cardiorenal physiology [9]. Correlations between GFR and aldosterone (r = −0.42, p < 0.01) and between GFR and ACE (r = −0.36, p < 0.01) support an association rather than a directional effect (Table 3). Prior literature links higher aldosterone to adverse cardiovascular outcomes in CKD and AMI populations [2,10-12], plausibly via sodium retention, hemodynamic load, and profibrotic signaling [13,14]. Our findings align with these observations at an associative level. By contrast, ANP showed no between-group difference, and vWF was numerically higher in the preserved-GFR group but was nonsignificant. These null results may reflect limited sensitivity of ANP/vWF to early renal function differences, competing physiology in AMI with HF (preload and acute endothelial activation), timing of sampling within 24 hours, and sample variability.

Because we did not measure renin or angiotensin II, inference about complete RAAS activation is limited and rests on partial markers (aldosterone/ACE) [15-17]. Moreover, serum ACE reflects circulating enzymatic activity and may not fully represent tissue-level RAAS biology (e.g., myocardial or renal ACE/Ang II signaling). Local RAAS compartments and alternative Ang II-generating pathways (e.g., chymase) can diverge from plasma measures; accordingly, ACE here functions as a surrogate rather than a direct readout of tissue RAAS activation, which limits mechanistic inference [15-17].

We note prior work on central mineralocorticoid receptor effects in cardiovascular regulation; however, as our study collected no CNS-specific data, we present this as background and a future research direction rather than as a mechanism inferred from our cohort [18-22].

Clinical implications should be considered exploratory. External evidence supports RAAS-targeting strategies in related cardiorenal populations [5,23-25], including randomized trials of non-steroidal MR antagonism in diabetic CKD (FIDELIO-DKD, FIGARO-DKD) [24,25]; however, our cross-sectional analysis does not provide interventional or prognostic confirmation. Any potential therapeutic relevance for AMI with reduced GFR requires prospective longitudinal and interventional trials to determine whether biomarker modulation translates into improved outcomes [26]. These findings provide insight into the association between renal function and RAAS-related biomarkers in AMI. While prior studies link RAAS dysregulation to adverse cardiovascular outcomes [2,10-12], our analysis did not assess clinical outcomes, and the observed relationships should be interpreted as associations rather than prognostic effects.

Limitations

The cross-sectional design precludes causal inference; this was a single-center study with a narrow age range (40-60 years), limiting generalizability. This study uses a historical cohort (2007-2011). We did not perform multivariable adjustment for potential confounders (e.g., medication use, blood pressure, HF severity, comorbidities), and subgroup analyses by CKD stage, sex, or etiology were not powered. RAAS characterization relied on aldosterone and serum ACE only; the absence of renin and angiotensin II and of tissue-level assessments constrains interpretation of RAAS activation [15-17]. We did not capture in-hospital or post-discharge outcomes; thus, no prognostic comparisons between GFR groups can be made from these data. Biomarker sampling occurred within 24 hours of presentation, which may not capture later neurohumoral dynamics. While core RAAS and endothelial mechanisms are biologically stable, evolving AMI/CKD management since accrual limits direct extrapolation; therefore, findings are hypothesis-generating and should be interpreted in the context of contemporary practice standards [6,8]. Finally, our biomarker panel did not include other relevant systems, such as sympathetic activation (e.g., plasma norepinephrine), the endothelin axis (e.g., endothelin-1), or inflammatory mediators (e.g., interleukin-6). Incorporating these markers in future studies could refine mechanistic interpretation and strengthen external validity.

Medication data were unavailable: exposure to ACEi/ARB/mineralocorticoid receptor antagonists was not documented or followed, and these agents can influence aldosterone and ACE levels; therefore, residual confounding cannot be excluded. Our analysis did not collect longitudinal outcomes; therefore, links to adverse outcomes are drawn from external literature and should not be inferred from our data.

In summary, lower GFR in AMI was associated with higher aldosterone and ACE, consistent with RAAS-related imbalance. Given the nominal between-group differences, incomplete RAAS profiling, and absence of outcomes, these results should be viewed as hypothesis-generating. Future work should apply KDIGO-aligned GFR strata with sufficient sample size, include broader neurohumoral and inflammatory panels (renin/Ang II, norepinephrine, endothelin-1, IL-6), and use longitudinal designs to clarify prognosis and test whether targeted interventions improve clinical outcomes [5,23-26].

## Conclusions

In this single-center, cross-sectional, exploratory analysis of AMI patients (ages 40-60), lower GFR (≤90 mL/min) was associated with higher aldosterone and ACE, whereas ANP showed no between-group difference, and vWF was numerically higher in the preserved-GFR group but not significant. Between-group differences were nominal and did not meet our prespecified p < 0.01 threshold; accordingly, these results indicate association rather than causality or prognosis. The ≤90 vs >90 mL/min split was used to sample a renal-function continuum and is not a CKD definition (KDIGO classifies 60-89 mL/min/1.73 m² as mildly decreased GFR, not diagnostic of CKD without additional markers). Because longitudinal outcomes and predictive performance (e.g., receiver operating characteristic/area under the curve [ROC/AUC]) were not assessed, aldosterone and ACE should be viewed as candidate markers rather than established “early indicators.”

Any therapeutic relevance of RAAS blockade in this context is supported by external evidence in related populations and not demonstrated by our dataset. Generalizability is limited by the single-center setting, selective cohort characteristics, and narrow age range, and residual confounding is possible given unmeasured factors (e.g., medication exposure, HF severity, blood pressure, comorbidities). Overall, these findings are hypothesis-generating and should be interpreted alongside contemporary practice standards.

Priorities for future work include KDIGO-aligned strata with adequate power; longitudinal trajectories of a broader neurohumoral/inflammatory panel (renin/Ang II, endothelin-1, norepinephrine, IL-6) alongside aldosterone/ACE; multivariable adjustment for confounding; evaluation of incremental prognostic value (ROC/AUC, reclassification); and interventional trials to test whether biomarker modulation improves outcomes in AMI with impaired renal function.
